# Satellite Glial Cells Surrounding Primary Afferent Neurons Are Activated and Proliferate during Monoarthritis in Rats: Is There a Role for ATF3?

**DOI:** 10.1371/journal.pone.0108152

**Published:** 2014-09-23

**Authors:** Diana Sofia Marques Nascimento, José Manuel Castro-Lopes, Fani Lourença Moreira Neto

**Affiliations:** 1 Departamento de Biologia Experimental, Centro de Investigação Médica (CIM), Faculdade de Medicina do Porto, Universidade do Porto, Porto, Portugal; 2 Morphophysiology of the Somatosensory System Group, Instituto de Biologia Molecular e Celular (IBMC), Porto, Portugal; Massachusetts General Hospital/Harvard Medical School, United States of America

## Abstract

Joint inflammatory diseases are debilitating and very painful conditions that still lack effective treatments. Recently, glial cells were shown to be crucial for the development and maintenance of chronic pain, constituting novel targets for therapeutic approaches. At the periphery, the satellite glial cells (SGCs) that surround the cell bodies of primary afferents neurons in the dorsal root ganglia (DRG) display hypertrophy, proliferation, and activation following injury and/or inflammation. It has been suggested that the expression of neuronal injury factors might initially trigger these SGCs-related events. We then aimed at evaluating if SGCs are involved in the establishment/maintenance of articular inflammatory pain, by using the monoarthritis (MA) model, and if the neuronal injury marker activating transcriptional factor 3 (ATF3) is associated with these SGCs' reactive changes. Western Blot (WB) analysis of the glial fibrillary acidic protein (GFAP) expression was performed in L4-L5 DRGs from control non-inflamed rats and MA animals at different time-points of disease (4, 7, and 14d, induced by complete Freund's adjuvant injection into the left hind paw ankle joint). Data indicate that SGCs activation is occurring in MA animals, particularly after day 7 of disease evolution. Additionally, double-immunostaining for ATF3 and GFAP in L5 DRG sections shows that SGCs's activation significantly increases around stressed neurons at 7d of disease, when compared with control animals. The specific labelling of GFAP in SGCs rather than in other cell types was also confirmed by immunohistochemical labeling. Finally, BrdU incorporation indicates that proliferation of SGCs is also significantly increased after 7 days of MA. Data indicate that SGCs play an important role in the mechanisms of articular inflammation, with 7 days of disease being a critical time-point in the MA model, and suggest that ATF3 might be involved in SGCs' reactive changes such as activation.

## Introduction

Inflammation of the joint is characterized, among others, by debilitating mechanical hyperalgesia and persistent pain at rest. It is one of the major causes of chronic pain and therefore a relevant clinical problem in need of better therapeutic approaches. In spite of the great advances in the study of articular inflammatory painful conditions and the existence of reliable experimental models, the nociceptive neuronal mechanisms behind these pathologies are still vague and lack investigation [1].

In the peripheral nervous system (PNS), pain mechanisms involve sensitization of primary afferents neurons whose cell bodies are located in the dorsal root ganglia (DRG). In fact, the thermal and mechanical sensations captured at the skin, viscera and joints are conveyed into the CNS through the DRGs, implying that they are the first relay centers for sensory input transmission from periphery [2] and an important site for the processing of neural information [3].

In the DRGs, the cell bodies of these primary afferents are anatomically surrounded by satellite glial cells (SGCs) forming distinct functional units [4]. SGCs may be identified by the expression of several glial markers such as glutamine synthetase (GS) and S100β. The immunoreactivity against glial fibrillary acidic protein (GFAP), an intermediate filament protein, is not readily detectable in SGCs at a resting state or under normal physiological conditions. However, following nerve injury, inflammation or viral infection, GFAP becomes detectable in the SGCs that become activated by the pathological insult. Thus, in the PNS, GFAP expression is commonly used as a marker of SGCs activation [4]–[6]. Although SGCs' properties and functions have not yet been fully studied, it is now clear that these cells take an important part in the “intercellular communication” [3] with the neuronal cells they are in contact with.

The role of SGCs has been underestimated for a long time [7], but the available data reveal that they are important in the establishment and maintenance of pathological conditions, largely contributing to the development of chronic pain states. In fact, the SGCs' unique localization around neuronal cell bodies allows a bidirectional crosstalk [4] known to strongly influence nociceptive processing [3], [8]. Thus, under a pathological condition, neurons are known to release specific mediators, such as ATP, nitric oxide, and neuropeptides as calcitonin gene-related protein (CGRP) and substance P, that are able to activate SGCs. Activated SGCs may also release pro-inflammatory agents that contribute to continued neuronal sensitization [9]. There is also strong evidence pointing to the occurrence of morphological and biochemical changes in SGCs as a response to pathological conditions. Accordingly, both activation [11], [12] and proliferation [7], [13] of these cells have been described as a response to nerve injury and/or inflammation, and consequent pain development. However, the exact factors and the associated mechanisms leading to these reactive morphological and biochemical changes in SGCs, during a pathological condition, are still partially unknown. Additionally, the onset of those alterations in relation to disease progression has not either been thoroughly investigated in the majority of the studies.

Using a model of chronic articular inflammatory pain, the monoarthritis (MA) induced by Complete Freund's Adjuvant (CFA) injection in the tibiotarsal joint, we investigated if SGCs might also be playing a role in this pathological condition. In order to evaluate SGCs activation, we quantified GFAP expression in the DRGs of MA animals by Western Blot (WB). We also confirmed by immunohistochemistry (IHC) that GFAP expression is specifically occurring in SGCs. To evaluate the time course pattern of such changes in relation to the progression of the inflammatory condition we used different time-points of the disease (4, 7 and 14d after CFA injection), that allowed us to correlate the data with our previous studies in the same pain model [14]. We have previously found a significantly increased expression of the neuronal injury marker activating transcriptional factor 3 (ATF3) in the DRGs at the initial time-points of MA [14], with a peak of expression at day 4, which suggested that some degree of neuronal damage is occurring in the early stages of this disease. Moreover, it has been suggested that the expression of injury factors might trigger part of the neuron-SGCs communication events [15]. Thus, with the aim of evaluating if activation of SGCs occurs preferentially around damaged/stressed neurons, we also performed co-immunolabeling assays for GFAP and ATF3 in the DRGs of controls and MA animals. Lastly, we also analyzed the incorporation of bromodeoxyuridine (BrdU) as a way to investigate if proliferation of SGCs is also occurring during MA.

## Materials and Methods

### Animal handling and Monoarthritis (MA) induction

All the procedures were carried out according to the European Communities Council Directive of September 22, 2010 (2010/63/EC) and to the ethical guidelines for investigation of experimental pain in animals [16], and were authorized by the animal welfare body (ORBEA) of the Faculty of Medicine of the University of Porto, where the experiments were performed. Animals used for Western Blot (WB) purposes (section 2.3) were decapitated after light volatile anesthesia with isoflurane. Those animals that were perfused through the ascending aorta for IHC assays (section 2.4), were deeply anesthetized with sodium pentobarbital. The humane endpoints defined for this project were always respected. Efforts were made in order to minimize pain and distress and reduce the number of animals used. Experiments were carried out in a total of 44 adult male Wistar rats (Charles River Laboratories, France) weighing between 200 and 300 g. Animals were housed 2–3 animals per cage under controlled conditions of lighting (12 h light/12 h dark cycle) and temperature as well as water and food *ad libitum*.

Monoarthritis (MA) was induced by injecting 50 µL of complete Freund's adjuvant (CFA), into the left tibiotarsal joint [17] under isoflurane anesthesia (5% for induction, 2.5% for maintenance). The CFA solution (5,45 mg/mL) was prepared as previously described [18] and monoarthritic animals were sacrificed at 4, 7 or 14 days of inflammation. Control (non-inflamed) animals were similarly injected with 50 µL of CFA vehicle and were allowed to survive for 2 days, as previously described [14]. Habituation of the animals to the experimenter was performed for several days before CFA injection and during the progression of MA, to minimize fear-motivated behaviors. The evolution of the inflammatory reaction was monitored daily and was scored taking in consideration the inflammatory signs of the injected ankle and reduction of the locomotor activity [19]. The severity of the inflammation was further evaluated by measuring the diameter of the animals' affected paw just before sacrifice [14]. One of the animals that had been injected with CFA to be used in the BrdU experiments developed polyarthritis, characterized by inflammatory signs in the contralateral non-injected paw and tail, as described before [17], and therefore was immediately excluded from the study.

### Bromodeoxyuridine (BrdU) administration

Bromodeoxyuridine (BrdU-B5002, Sigma-Aldrich) was intraperitoneally (i.p.) injected (50 mg/Kg of animal weight) immediately after the preparation of a solution of 50 mg/mL, 10% in dimethyl sulfoxide (DMSO) [8]. Injections were performed twice daily, beginning at the day of CFA or CFA vehicle intra-articular injection (day 0), until 24 h prior to animals sacrifice (to allow BrdU clearance) by intracardiac perfusion, as described below. The following experimental groups were used: controls (CFA-vehicle non-inflamed rats injected with BrdU until day 3 and sacrificed at day 4; N = 6 rats), 4d MA (CFA-inflamed rats injected with BrdU until day 3 and sacrificed with 4d of disease; N = 5 rats) and 7d of MA (CFA-inflamed rats injected with BrdU until day 6 and sacrificed with 7d of disease; N = 6 rats). Prior to these experiments, a group of naive animals was injected twice a day, with 10% DMSO solution i.p., for 6 days and no toxic effects or signs of peritoneal inflammation were found (data not shown).

### Analysis of GFAP expression by Western Blotting

In order to investigate SGCs activation, the expression of glial fibrillary acidic protein (GFAP) was evaluated by WB analysis of freshly harvested DRGs from MA (with 4, 7 and 14 days of disease, N = 5 animals per group) and control animals (N = 6) that had been sacrificed by decapitation under light anesthesia with isoflurane. To correlate data with the previous studies [14], DRGs from 2d MA animals were also analyzed but significant changes were not found (data not shown). Thus, this time-point was excluded from the following experiments.

For each animal, the L4 and L5 ganglia were pooled, separately for the ipsi and contralateral sides, and then were lysed and homogenized in 70 µL of radio immuno precipitation assay (RIPA) buffer containing sodium chloride 150 mM, triton X-100 1%, sodium deoxycholate 0.5%, sodium dodecyl sulphate (SDS) 0.1% and Tris pH 8.0 50 mM. Cocktails of protease and phosphatase inhibitors (1∶100, Sigma-Aldrich P8340, P5726 and P0044) were also added to the buffer. The samples were sonicated and centrifuged (20 minutes at 20,000 g), the pellets were discarded and the supernatants were used for analysis. The proteins were quantified by the bicinchoninic acid (BCA) protein assay. After heating at 94°C, 30 µg of protein were loaded for each lane and separated on 14% sodium dodecyl sulphate-polyacrylamide (SDS/PAGE) gels. The proteins were then transferred into nitrocellulose membranes which were blocked with non-fat milk (5% milk powder diluted in tris buffer saline tween20; TBST buffer), for one hour, at room temperature, to prevent non-specific bindings. In order to detect GFAP, the membranes were incubated in monoclonal mouse anti-GFAP antibody (Mab360, Chemicon-Millipore) diluted 1∶500 in TBST with 2% of normal goat serum (NGS), for 24 hours at 4°C. As a loading control, the detection of β-actin (polyclonal rabbit anti-β-actin antibody, Ab8227 ABCAM, Cambridge, UK) diluted 1∶4000 in TBST with 2% of normal horse serum (NHS) was also performed.

Detection of GFAP was achieved by incubation in goat anti-mouse secondary antibody conjugated with horseradish peroxidase (HRP; sc-2031, Santa Cruz Biotechnology, Inc), diluted 1∶5000 in TBST with 5% milk powder, for 1 hour, at room temperature. β-actin was also detected using a donkey anti-rabbit secondary antibody conjugated with HRP (711-035-152, Jackson Laboratories), diluted 1∶5000 in TBST with 5% milk powder. Antibody binding was visualized with the SuperSignal West Pico Chemiluminescent Substrat kit (Thermo Scientific; 34080) and the bands were detected by exposing the membranes to X-ray films (KODAK XOMAT Blue (XB) Film, Perkin Elmer, USA; NEF586001EA). Each blot, containing independent samples, was run in triplicates and means were used as raw values.

### Double Immunohistochemistry against GFAP-ATF3 or BrdU-GS

After deep anesthesia with sodium pentobarbital (Eutasil, Ceva, Sante Animale, France; i.p., 75 mg/kg of animal body weight), the animals were perfused through the ascending aorta with 250 mL of oxygenated Tyrode's solution followed by 750 mL of paraformaldehyde (PFA) 4% in phosphate buffer saline 0.1 M (PBS 0.1 M). The ipsi- and contralateral DRGs corresponding to spinal segment L5 were removed and post fixed in the same fixative solution for 4 h and then cryoprotected over night (in sucrose 30% in phosphate buffer 0.1 M). The DRGs were cut into 14 µm sections in a freezing cryostat (−20°C). The tissue was collected sequentially into 5 different poly-L-lysine coated slides, was air dried and stored at −20°C until immunohistochemistry was performed.

To confirm if activation of SGCs is possibly occurring in cells surrounding damaged/stressed neurons (ATF3-positive profiles), double immunoreactions against GFAP and ATF3 were performed. Each slide (containing every fifth section of each L5 DRG) from both controls non-inflamed and 7d MA animals was first thawed and washed in PBS 0.1 M and then PBS containing 0.3% Triton X-100 (PBST). In order to avoid unspecific bindings, sections were incubated for 1 hour in a blocking solution containing 10% of NGS in PBST. Afterwards, slides were incubated for 48 h at 4°C in the primary antibodies rabbit anti-GFAP (ab7260, Abcam, 1∶1000), and mouse anti-ATF3 (ab58668, Abcam, 1∶200), diluted in PBST containing 2% of NGS. After several washes in PBST with 2% of NGS, slides were finally incubated, for 1 hour, at room temperature, in goat anti-rabbit 568 (A11011, Molecular Probes, 1∶1000) and donkey anti-mouse 488 (A21202, Molecular Probes, 1∶1000) secondary antibodies diluted in a solution of PBST with 2% of NGS.

To evaluate SGCs proliferation, sections from perfusion-fixed L5 DRGs of control non-inflamed, 4d and 7d MA animals, previously injected with BrdU, were double immunoreacted against BrdU (which marks proliferating cells) and GS. Slides containing every fifth section of each DRG were treated following a protocol similar to that described above, except that slides were firstly incubated in HCl at 60°C for 30 minutes and then 5 minutes in Borax 0.1 M, for antigen retrieval. Blocking was done in a solution of 10% normal swine serum (NSS) in PBST with 7.5 mg/mL of glycine. Slides were afterwards incubated in sheep anti-BrdU (BP2295, Acris, 1∶100) and mouse anti-GS (MAB302, Millipore, 1∶500), in a PBST solution with 2% of NSS. Detection was achieved by incubation in a biotinilated donkey anti-sheep secondary antibody (B-7390, Sigma Aldrich), 1∶200 diluted in PBST with 2% of NSS, for 1 hour at room temperature. After thorough washes in PBST, the slides were incubated in streptavidin 488 (S32354, Molecular Probes) and Alexa 568 donkey-anti-mouse (A10037, Molecular Probes), both 1∶1000 in PBST with 2% of NSS.

After the immunoreaction, the slides with the stained sections were stored in PBS 0.1 M at 4°C until they were mounted for visualization under a fluorescent microscope. For microscopic analysis, the slides were coverslipped with a mounting media (solution containing 3 parts of glycerol and 1 part of PBS 0.4 M).

### Data analysis

#### Quantification of band intensity in Western Blotting

The protein levels were obtained by densitometric analysis of the signal intensity in the blots, in pixels, using the image computer software ScionImageR (Scion Corporation). Both the areas of the lanes and the background signal were used for values normalization. β-actin was used as loading control and a ratio between GFAP/β-actin protein levels was calculated. Additionally, ratios between the ipsi and contralateral levels were calculated for comparison between the different MA groups and controls. The assays were typically performed three times on samples obtained from independent groups of rats and means of these triplicates were used as raw values.

#### Immunoreactivity detection and cell counting

The immunohistochemistry analysis was performed by using a fluorescence microscope (AXIO Imager.Z1, Zeiss), coupled to a digital camera (Axiocam MRm) and a computer image software (Axiovision 4.6) to grab the images. For the photomicrographs the acquisition conditions, such as amplification of the objective, light intensity, contrast and hue, were maintained constant.

The expression of GFAP in SCGs was confirmed by immunodetection. SCGs were distinguished from nerve cell soma and other perineuronal cells by their shape, position, orientation and nuclear characteristics [20]. Neurons surrounded by GFAP-positive SGCs in half or more than half of their circumference were assumed as positive neuronal profiles. The total number of these immunolabeled GFAP-positive neuronal profiles (**GFAP^+^_total_ NP**) was quantified. The total number of cells bodies of primary afferents analyzed was defined here as **NP_total_** and counted for each slide (corresponding to an animal and containing every fifth section of each L5 DRG). For normalization **GFAP^+^_total_ NP** was divided by **NP_total_** (**GFAP^+^_total_ NP/NP_total_**), and presented as percentage.

The total number of double labeled neuronal profiles (GFAP-positive neuronal profiles also expressing nuclear ATF3; **Double^+^_total_**) was also counted and divided by the total number of analyzed neurons (**Double^+^_total_/NP_total_**), and the final value is presented as percentage. Additionally, we calculated the percentage of double labeled neuronal profiles in the total ATF3-positive population **(Double^+^_total_/ATF3^+^_total_**).

To evaluate the proliferation of SGCs, the total number of double-labeled cells against BrdU and GS (**SGCs^+^** **_total_**) was counted in each slide (containing every fifth section of each L5 DRG). For normalization, a ratio between **SGC^+^_total_/NP_total_** was calculated so that values of different animals could be compared. In order to calculate the mean of proliferating SGCs (**SGC^+^**) around neurons, we divided the **SGCs^+^** **_total_** by the total number of neuronal profiles surrounded by at least one positively labeled SGC (**Mean SGC^+^ around NP**) [7]. Neuronal profiles surrounded by SGC^+^ in half or more than half of their circumference were also counted and denominated as **NP^+^** [21]. Again, for means of standardization, a ratio between **NP^+^_total_/NP_total_** was calculated to allow comparison between different animals and experimental groups.

#### Statistical analysis

Statistical analysis was performed by using GraphPad Prism 5 (GraphPad Software) and SPSS 13.0. One-way analysis of variance (one-way ANOVA) was performed to investigate significant differences between the different experimental groups. For the WB data, ANOVA was followed by the Tukey's Multiple Comparison post-hoc test. In this case, the values were calculated as ratios between the ipsi and contralateral sides after normalization against the loading control, β-actin. Results were displayed as mean±SEM (N = 6 for controls; N = 5 for all the other experimental groups). Data from immunohistochemical GFAP detection was analyzed using ANOVA followed by the Bonferroni post-hoc test. Results (**GFAP^+^_total_;NP/NP_total_**) were shown as mean±SEM (N = 5 for all the experimental groups). The GFAP-ATF3 double-labeling data was analyzed using one-tailed Student's t-test analysis between the controls and 7d MA groups. Results (**Double^+^_total_/NP_total_** and **Double^+^_total_/ATF3^+^_total_**) were displayed as mean±SEM (N = 5 for 7d MA and N = 4 for controls). For BrdU quantification, ANOVA was followed by the Newman Keuls Multiple Comparison test, for all the three different displayed results. All values (**SGC^+^_total_/NP_total_**; **NP^+^_total_/NP_total_**; **Mean SGC^+^/NP**) were shown as mean±SEM (N = 6 for controls and 7d MA; N = 5 for 4d MA). In all the statistical analyses, a level of significance of *P*<0.05 was assumed.

## Results

### SGCs are activated during MA

MA was successfully and homogenously induced in all the animals injected with CFA, as they were all showing severe inflammatory symptoms with swelling, redness and avoidance to put weight over the inflamed paw at each time-point of disease. This was reflected in mean inflammatory scores near 4 (maximum score), immediately after the second day of MA. This condition was maintained up to the 14th day, as well as increased paw volumes (data not shown), in accordance with our previous work [14]. Controls showed insignificant mean scores.

**Figure 1:**
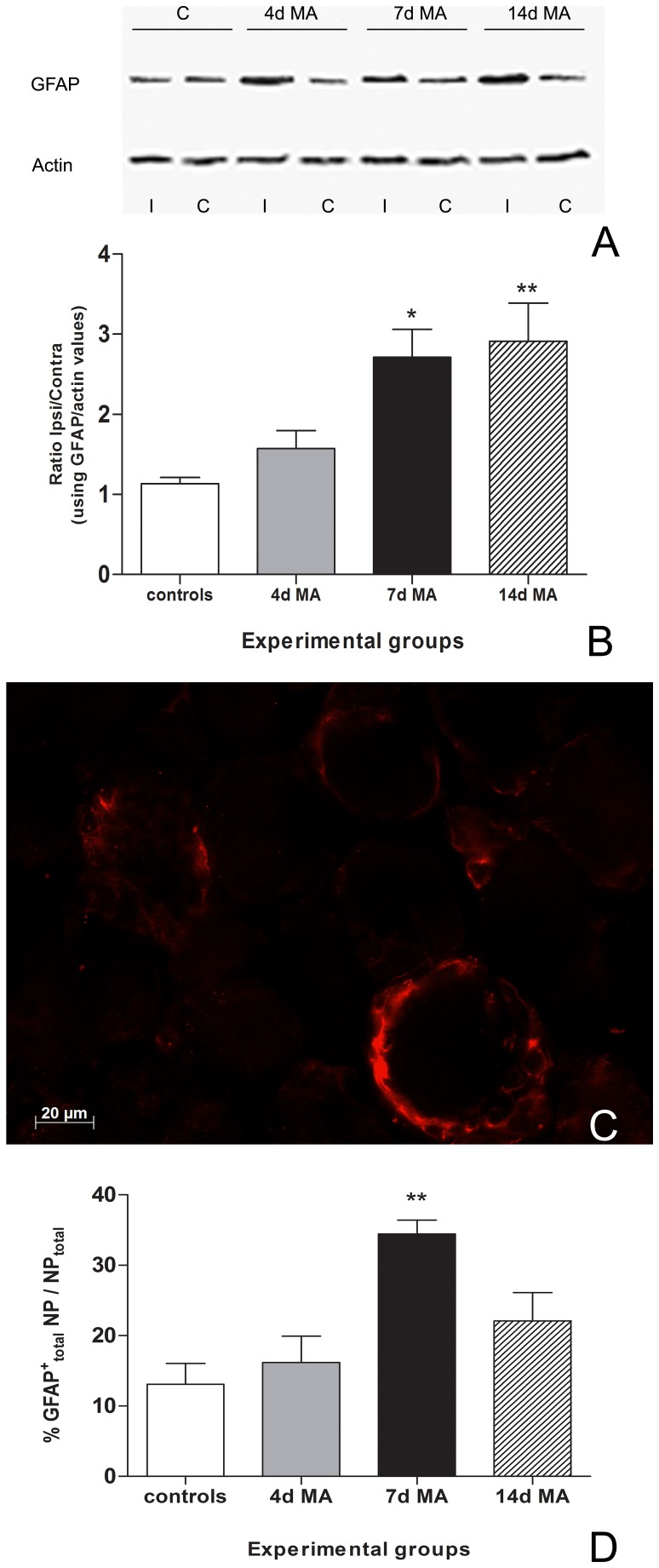
GFAP overexpression during MA. (**A**) GFAP levels in 4d, 7d and 14d MA animals were always higher in the ipsilateral DRGs (lines 3, 5, 7) when comparing to DRGs from the contralateral side (lines 4, 6, 8). As expected, control values were similar for both ipsi and contralateral sides (lanes 1 and 2). (**B**) The ratios between Ipsi and Contralateral GFAP levels (GFAP/actin values) were significantly increased at day 7 and 14 days of MA which suggests activation of SGCs at around 1 week after disease induction. (**C**) Single immunolabeling for GFAP (red) specifically in SGCs, in a L5 DRG from a 7d MA animal (bar represents 20 µm). **D**) The percentage of the total number of GFAP-positive neuronal profiles in the total neuronal population (**GFAP^+^_total_ NP/NP_total_**) significantly increases at 7d MA. All values are shown as Mean±SEM, In B) N = 6 for controls and N = 5 for all the other experimental groups. * represents p<0.05 relatively to controls. One-way ANOVA was followed by Tukey's Multiple Comparison post-hoc test. In D) N = 5 for all experimental groups.** represents p<0.01, relatively to controls. One-way ANOVA was followed by Bonferroni post-hoc test.

Western blot analysis showed that the GFAP levels in MA animals were always higher in the ipsilateral (lanes 3, 5, 7 of Fig. 1A) than in the contralateral DRGs (lanes 4, 6, 8 of Fig. 1A). Consequently, ratios between ipsi and contralateral GFAP levels were significantly increased at day 7 (2.71±0.35; p<0.05) and 14 of disease (2.91±0.47; p<0.01), when compared with controls (1.13±0.08) (Fig. 1A and B). Controls showed a non-significant basal expression in both ipsi- and contralateral sides, as expected.

In order to confirm that the GFAP expression detected by Western blot was actually occurring in SGCs, we immunoreacted perfusion-fixed L5 DRG sections of control, 4d, 7d and 14d MA animals against GFAP. The specific labeling, the morphology and the unique localization around the cell bodies of DRGs neurons confirmed that GFAP expression is actually occurring in SGCs (Fig. 1C) [4]. Quantification of the total number of positive GFAP neuronal profiles (**GFAP^+^_total_ NP/NP_total_**) revealed that there are significant increases for 7d MA animals (34.45±1.95%; p<0.01) when compared with controls (13.09±2.95%) (Fig. 1D). Animals with 14d MA presented also an increased number of GFAP-positive neuronal profiles in comparison to non-inflamed controls, although statistical significance was not achieved (Fig. 1D).

### Activation of SGCs increases around stressed neurons, in MA

**Figure 2:**
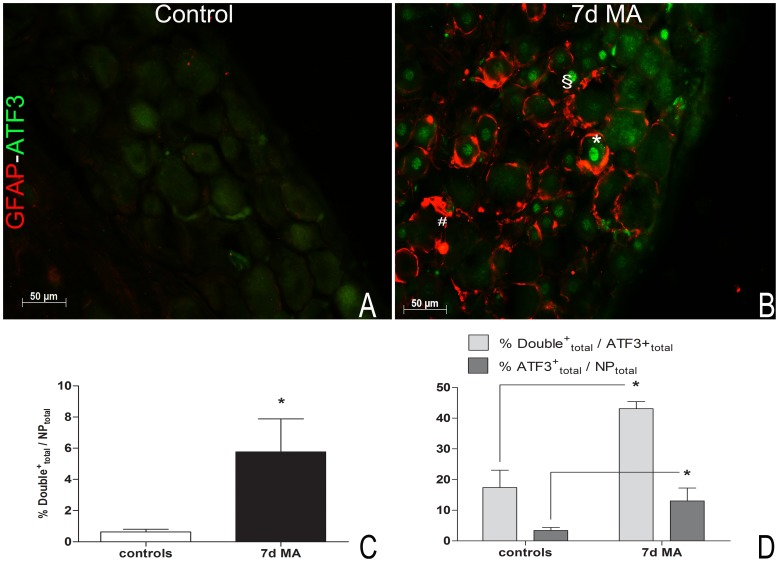
GFAP labeling in SGCs surrounding ATF3 positive neurons increases at 7d MA. (**A, B**) Double labeling for ATF3 (green) and GFAP (red), in L5 DRGs sections from a control (**A**) and a 7d MA animal (**B**). (**C**) The percentage of double labeled neuronal profiles in the total neuronal population (**% Double^+^_total_/NP_total_**) increases at 7d MA. (**D**) The percentage of double labeled neuronal profiles in the total ATF3-positive neuronal population (**Double^+^_total_/ATF3^+^_total_**) also increases after 7d MA, even though ATF3-positive neurons represent a small portion of the total neuronal population of the DRG (**%ATF3^+^_total_/NP_total_**). In A and B, the bar represents 50 µm, ^#^ identifies a single labeled GFAP-positive neuronal profile, ^§^ identifies a single labeled ATF3-positive neuron and* identifies co-labeling of both GFAP and ATF3. In C and D, all values are shown as Mean±SEM with N = 5 for 7dMA and N = 4 for controls. * Represents p<0.05 relatively to control animals. One-tailed Student's t-test analysis.

The total number of neurons counted as positive for both ATF3 and GFAP (**Double^+^_total_/NP_total_**) (Fig. 2A and B) was significantly increased at 7 days of MA (5.76±2.12%) when compared with controls (0.63±0.18%, p<0.05) (Fig. 2C). Also, the percentage of double labeled cells in the total ATF3-positive neuronal population **(Double^+^_total_/ATF3^+^_total_**) increased at 7d MA (43.09±2.37%, p<0.05) in comparison with controls (17.38±5.68%).

### SGCs proliferate during MA

**Figure 3:**
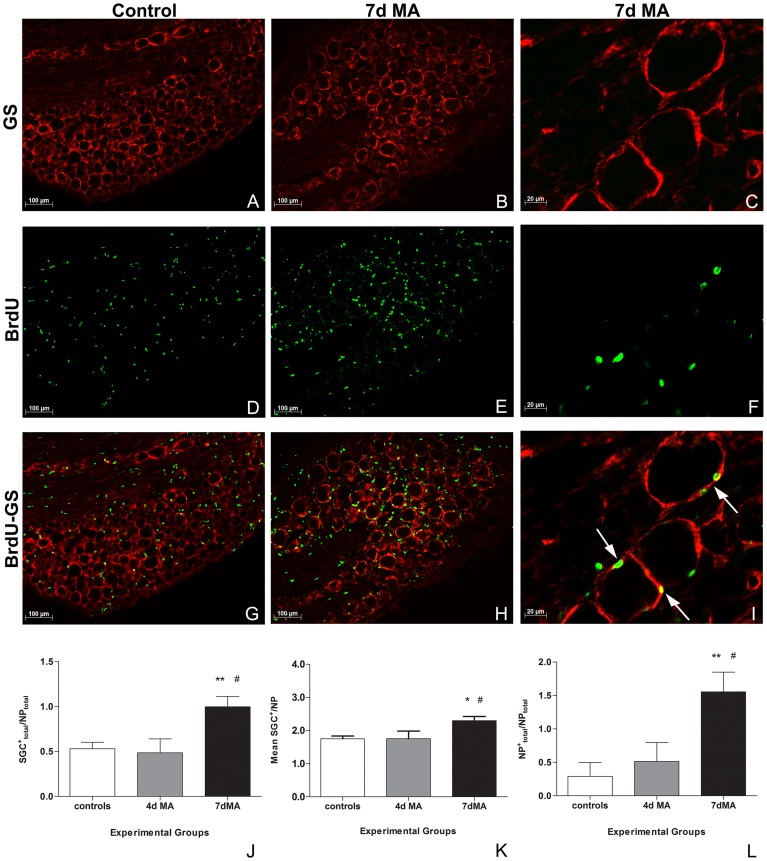
BrdU incorporation increases during MA. (**A–I**) Immunofluorescence labeling for GS (red) (**A, B, C**), BrdU (green) (**D, E, F**) and respective colocalization between both (**G, H, I**), in a L5 DRG of a control and a 7d MA animal (bar represents 100 µm). **A**rrows point to well visible double-labeled SGCs. An amplified image from a L5 DRG of a 7d MA animal shows BrdU labeling in detail (bar represents 20 µm) (**C, F, I**). (**J**) The number of proliferating SGCs (SGCs^+^), in the total number of neuronal profiles (**SGC^+^_total_/NP_total_**), significantly increases at 7d MA. (**K**) The mean number of proliferating SGCs around a specific neuron (**Mean SGC^+^/NP**) also increases at 7d MA. (**L**) The number of positive neuronal profiles (**NP^+^_total_/NP_total_**) is also significantly higher in 7d MA, when compared with both control non-inflamed and to 4d MA animals. All values shown as Mean±SEM. N = 6 for controls and 7d MA, and N = 5 for 4d MA experimental group.* Significant differences relatively to control. # Significant differences relatively to 4d MA.* or ^#^ represents p<0.05; ** represents p<0.01. One-way ANOVA was followed by Newman-Keuls Multiple Comparison post-hoc test.

BrdU was injected in controls (non-inflamed) and in 4 and 7d MA animals (Fig 3D, E, F). In order to confirm BrdU incorporation in SGCs, a double immunocolocalization with GS was performed (Fig. 3A, B, C for GS immunoreactivity; Fig. 3G, H, I for colocalization of BrdU with GS). The **SGC^+^_total_/NP_total_** significantly increased at 7d of MA (1.00±0.11), when compared with both controls (0.53±0.07; p<0.01) and 4d MA (0.49±0.15, p<0.05) animals (Fig 3J and Table 1). Not only the overall number of SGC^+^ increased in the ganglia along disease progression, but, in addition, the number of proliferating SGCs around a specific neuron also augmented. In fact, the **Mean SGC^+^ around NP** was also significantly higher at 7d MA (2.30±0.13) than in controls (1.75±0.08; p<0.05) and 4d MA (1.75±0.23; p<0.05) animals (Fig 3K and Table 1). Moreover, as the number of proliferating SGCs around a neuron increased, more positive neuronal profiles were also found. Thus, **NP^+^_total_/NP_total_** was also significantly higher in 7dMA (1.55±0.29) than in controls (0.29±0.21; p<0.01) and 4d MA (0.51±0.28; p<0.05) (Fig 3L and Table 1). In summary, in all three types of quantification, the controls and 4d MA animals showed very similar values, both being statistically different from 7d MA (Table 1).

**Table 1:** SGCs significantly proliferate at 7d of MA.

	Total SGCs^+^_total_/NP_total_	Mean SGCs around NP	% (NP^+^_total_/NP_total_)
**Controls**	0.53±0.07	1.75±0.08	0.29±0.21
	**(6485/12715)**		**(21/12715)**
**4dMA**	0.49±0.15	1.75±0.23	0.51±0.28
	**(4401/9117)**		**(28/9117)**
**7dMA**	1.00±0.11**^#^	2.30±0.13*^#^	1.55±0.29**^#^
	**(13500/14048)**		**(212/14048)**

Significant increases in the total number of proliferating SGCs (**SGC^+^_total/_NP_total_**), in the mean number of SGC^+^ surrounding a specific NP (**Mean SGC^+^ around NP**), and in the total number of positive neuronal profiles (NP surrounded by half or more than half of their circumference by SGC^+^ - **NP^+^_total_/NP_total_**), were found at 7dMA.

Values shown as Mean±SEM. In brackets, the total number of cells analyzed for each ratio is displayed. N = 6 for controls and 7d MA; N = 5 for 4d MA. * Significant differences relatively to controls. # Significant differences relatively to 4d MA.* or # represents p<0.05; ** represents p<0.01. One-way ANOVA followed by Newman-Keuls Multiple Comparison post-hoc test.

## Discussion

In this study, we show for the first time in the CFA-induced monoarthritis model of chronic joint inflammation that SGCs are activated and proliferate, with a specific temporal profile. Moreover, significant increases in the GFAP labeling in activated SGCs surrounding ATF3 positive (stressed) neurons were also found. This fact suggests that neuronal ATF3 might be involved in the reactive biochemical and morphological changes occurring in SGCs during a chronic pathological state.

Western blot analysis showed that GFAP levels in the ipsilateral DRGs of MA rats are higher than in the contralateral ganglia, and that this ipsi/contra ratio is significantly increased at 7 and 14 days of disease induction, when compared with control non-inflamed animals. Immunohistochemical quantification of GFAP-positive neuronal profiles in the sections of L5 DRGs also showed significantly increased levels at 7 days of MA. At 14d of MA, although statistical significances were not found, the values were still higher than in controls. The slight differences between WB and IHC data at 14 days of MA are certainly due to the distinct methodological approaches. In the WB assay we measured the total amount of protein in the whole DRGs, which contain both neurons, SGCs and Schwann cells. It is possible that Schwann cells, that also express GFAP [22]–[24], have a small contribution to the proteic levels measured in the WB. On the other hand, the IHC data represent the number of neurons surrounded by GFAP-positive SGCs, and it is unlikely that this quantification has been biased by considerable Schwann cells' contribution since these cells are morphologically distinct from SGCs. Thus, altogether the data from these two different experiments indicate that SGCs are significantly activated after 7days of MA and at least until 2 weeks of disease induction, and that the number of positive neuronal profiles increases around day 7, suggesting a higher number of sensitized neurons. In fact, activated SGCs are known to release several pro-inflammatory and other mediators that promote neuronal sensitization [9], [25]. Accordingly, it is expectable that neurons surrounded by a higher number of activated SGCs are also in a higher level of excitability [3], [4], [10].

These data are in accordance with several recent studies proposing that, after peripheral injury and/or inflammation, SGCs undergo relevant reactive biochemical and phenotypic changes (such as activation, proliferation and hypertrophy) that might be related to the establishment/maintenance of certain pathological and painful states [6], [9], [11], [26]–[28]. In fact, GFAP expression was found to be increased in inflamed DRGs, at 7 days of model induction (chromic gut suture application onto the DRG) [6], as well as in the trigeminal ganglia of rats with orofacial inflammatory pain [27]. Additionally, two days post-CFA injection into the whisker pad area, the mean percentage of trigeminal ganglia neurons encircled by GFAP and IL-1beta-immunoreactive cells was significantly increased compared with controls [25]. These data corroborate with our results for the GFAP expression in MA animals and indicate that the first week of disease progression seems to be crucial for the events associated to SGCs activation. The slight differences in the temporal expression pattern of GFAP are probably due to the pathophysiological differences of the models under study. As observed, SGCs activation occurs in the initial time-points of disease progression in inflammatory conditions, while little is known about the more prolonged time-points [6], [25]–[27]. Conversely, it seems that nerve damage provokes a more demarked and prolonged effect on SGCs activation. Indeed, in neuropathic pain models, such as in chemically-induced neuropathy, GFAP levels were also significantly higher after 1 week, followed by a decrease to control values only 1 month later [28]. In the spinal nerve ligation (SNL) neuropathy model, GFAP expression increased immediately after 4 hours, gradually increasing up to 7 days and staying high until the end of the experiment at day 56 [21]. In our studies, we observed that the activation of SGCs is significantly higher than in non-inflamed animals at least until 14 days of MA. We have previously proposed the occurrence of a neuropathic component in MA, possibly triggered by the initial inflammatory milieu at the joint cavity [14]. Actually, we reported that ATF3, a neuronal injury marker, is induced in primary afferent neurons, with a peak of expression at 4 days of MA [14], a fact that has not been described frequently in studies using other inflammatory models [1], [29], [30]. Therefore, the fact that neuronal damage is possibly occurring during MA, might be one of the reasons for the still significantly increased GFAP levels that we found at day 14. For time-points of disease evolution longer than this it is hard to speculate since the information available in the literature is limited. However, it is possible that GFAP levels do not remain high for too long, as it happens in a neuropathy, since MA is still a model triggered by an inflammatory insult.

Many studies are nowadays devoted to the identification of possible inducers of SGCs activation, in different conditions. Recently, some authors suggested a novel mechanism mediated by fractalkine as the trigger for SGCs' activation in the carrageenan-induced inflammation model [26]. Many other molecules were shown to be released by neurons with their receptors being found in SGCs [31], [32], therefore constituting possible mediators in neuron-glia crosstalk and triggers of SGCs' activation. Some authors also suggested that the expression of injury factors in stressed neurons might be one possible trigger for the activation and proliferation of SGCs as well as for the augmented intraganglionar communication [7]. Considering our previous data [14], we asked if ATF3 could be one of the injury factors involved in SGCs activation and in communication within neurons, during MA. Interestingly, we also found significant increases in the number of ATF3 positive neurons surrounded by GFAP-positive cells, in both the total neuronal (**Double^+^_total_/NP_total_**) and ATF3-positive populations (**Double^+^_total_/ATF3^+^_total_**), at 7d MA, which supports our hypothesis of a possible role of neuronal ATF3 in the reactive changes occurring in SGCs during articular inflammation. After 7d of MA, more than 40% of the ATF3-positive neurons were surrounded by GFAP-positive SGCs, even though the ATF3-positive population represents a small portion of all DRG neurons in the CFA-induced MA model, as we have previously described [14]. Our data are in accordance with other studies showing that the number of ATF3-immunoreactive (IR) neurons enclosed by GFAP-IR SGCs increased in a time-dependent manner in the maxillary nerve region of the trigeminal ganglia [12], in a model of molar extraction in the rat. Also, after chronic constriction injury of the infraorbital nerve, SGCs proliferation was observed preferentially around ATF3-positive neurons of the trigeminal ganglia, although GFAP expression was associated with both ATF3 IR and immunonegative neurons [13]. In a pathological condition, the number of gap junctions between SGCs increase and this phenomenon is intimately related to SGCs activation. Interestingly, gap junctions promote communication between adjacent SGCs enveloping neighboring neurons [3], [4], [10]. This might result in GFAP labeling around adjacent ATF3-negative neurons, suggesting that it is highly possible to have activated SGCs surrounding non-stressed neurons. Indeed, Gunjigake *et al*. also demonstrated in the model of rat molar extraction that SCGs' activation spread to uninjured neurons in the maxillary nerve region, as well as to the mandibular nerve region [12]. In these studies, as it happened in our case, it has been shown that there is a basal expression of GFAP in control animals, which is probably not labelling activated SGCs [12], [33]. Yet, the fact that these increases in GFAP labeling around ATF3 positive neurons are statistically different at 7 days of MA points to a possible relation between ATF3 expression and SGCs-related events.

In the MA animals, the number of SGCs proliferating in the whole DRG was also significantly higher at day 7 of disease when comparing with both controls and 4d MA. Not only the overall number of BrdU-positive SGCs in the DRG increased but also the number of SGCs proliferating around a specific neuron. Moreover, we found significantly more positive neuronal profiles in 7d MA animals, which is in accordance with other studies. There are few reports regarding the proliferation of SGCs, but early in the nineties other authors already showed that these cells proliferated after L5 nerve transection, with maximum activity of the incorporated radioactive marker 1 week after the model induction. In this case, proliferation started decreasing after this time-point [34]. Later, other BrdU incorporation studies showed that SGCs proliferate during Herpes Simplex virus infection, with increases up to 5 days of disease, the latest time point evaluated [35]. This was proposed to be part of a mechanism of neuronal survival during the disease [35]. The same group also found proliferation of SGC in an animal model of scarification of the skin, considered to be a model of minor tissue trauma [7]. BrdU incorporation increased by a 10 times fold 5 days after model induction, when compared with controls. Just recently, peaks of SGCs' proliferation were also observed nearly 4 days after model induction by chronic constriction injury of the infraorbital nerve [13]. Our results are in agreement with these previous studies, indicating that, also in MA, a significant proliferation of SGCs occurs. Also, they suggest that 7d of disease is a triggering time-point for this event.

The reactive changes observed in SGCs appear to be correlated with hypersensitivity to noxious stimuli, although the related mechanisms and their players still remain to be explored. In fact, it has been proved in several models that the administration of fluorocitrate, a metabolic inhibitor of SGCs, not only abolishes GFAP labeling in the DRGs but also alleviates pain [21], [26]. MA animals display increased allodynia and hyperalgesia in the ipsilateral paw, after 1 week of CFA injection, as we have already reported [36]. Therefore, it seems that the temporal profile of the biochemical changes found in the ipsilateral DRGs of these animals matches with the painful behavior. Although further studies are needed, data suggest that SGCs might be involved in the MA nociceptive mechanisms, as, in fact, found for other chronic pain models [21].

In summary, this study indicates that SGCs are not bystanders to MA, but that they are crucial in the mechanisms underlying articular inflammation. The reactive changes involving SGCs, namely their activation and proliferation, seem to be particularly active in the early phases of MA development, with peaks around the 7^th^ day, when the expression of the neuronal injury marker ATF3 is already subsiding, and allodynia and hyperalgesia are already obvious in the ipsilateral paws of inflamed animals. The exact functional implications of this early onset for the progression of the disease are still unknown. Additionally, ATF3 might be one potential target for the control of SGCs-mediated mechanisms. Thus, in the future, it will be important to unravel these key mechanisms which will be crucial for the development of new drugs targeting SGCs. This might help to overcome the inefficacy of certain pain-alleviating therapies [8], that have been traditionally devoted to target primary afferent neurons. This is highly relevant since pain associated with joint inflammatory diseases is still a challenge in the clinical practice.
